# Polymorphism in the *ELOVL6* Gene Is Associated with a Major QTL Effect on Fatty Acid Composition in Pigs

**DOI:** 10.1371/journal.pone.0053687

**Published:** 2013-01-14

**Authors:** Jordi Corominas, Yuliaxis Ramayo-Caldas, Anna Puig-Oliveras, Dafne Pérez-Montarelo, Jose L. Noguera, Josep M. Folch, Maria Ballester

**Affiliations:** 1 Departament de genètica animal, Centre de Recerca en Agrigenòmica (CRAG), Bellaterra, Spain; 2 Departamento de Mejora Genética Animal, Instituto Nacional de Investigación y Tecnología Agraria y Alimentaria (INIA), Madrid, Spain; 3 Genètica i Millora Animal, Institut de Recerca i Tecnologia Agroalimentàries (IRTA), Lleida, Spain; 4 Departament de Ciència Animal i dels Aliments, Universitat Autònoma de Barcelona (UAB), Bellaterra, Spain; University of Queensland, Australia

## Abstract

**Background:**

The *ELOVL fatty acid elongase 6* (*ELOVL6*), the only elongase related to *de novo* lipogenesis, catalyzes the rate-limiting step in the elongation cycle by controlling the fatty acid balance in mammals. It is located on pig chromosome 8 (SSC8) in a region where a QTL affecting palmitic, and palmitoleic acid composition was previously detected, using an Iberian x Landrace intercross. The main goal of this work was to fine-map the QTL and to evaluate the *ELOVL6* gene as a positional candidate gene affecting the percentages of palmitic and palmitoleic fatty acids in pigs.

**Methodology and Principal Findings:**

The combination of a haplotype-based approach and single-marker analysis allowed us to identify the main, associated interval for the QTL, in which the *ELOVL6* gene was identified and selected as a positional candidate gene. A polymorphism in the promoter region of *ELOVL6*, *ELOVL6:c.-533C>T*, was highly associated with the percentage of palmitic and palmitoleic acids in muscle and backfat. Significant differences in *ELOVL6* gene expression were observed in backfat when animals were classified by the *ELOVL6:c.-533C>T* genotype. Accordingly, animals carrying the allele associated with a decrease in *ELOVL6* gene expression presented an increase in C16:0 and C16:1(n-7) fatty acid content and a decrease of elongation activity ratios in muscle and backfat. Furthermore, a SNP genome-wide association study with *ELOVL6* relative expression levels in backfat showed the strongest effect on the SSC8 region in which the *ELOVL6* gene is located. Finally, different potential genomic regions associated with *ELOVL6* gene expression were also identified by GWAS in liver and muscle, suggesting a differential tissue regulation of the *ELOVL6* gene.

**Conclusions and Significance:**

Our results suggest *ELOVL6* as a potential causal gene for the QTL analyzed and, subsequently, for controlling the overall balance of fatty acid composition in pigs.

## Introduction

Food fatty acid (FA) composition is a critical aspect in human health and it is also relevant for meat quality. It determines important sensorial and technological aspects of meat due to the variability in the melting point of fatty acids. Thus, variation in fatty acids has an important effect on flavor, muscle color and firmness or softness of the fat in meat [1]. Meat fat is primarily composed of monounsaturated fatty acid (MUFA) and saturated fatty acid (SFA). Oleic acid is the most abundant and nutritionally relevant FA, followed by palmitic and stearic acids [2], [3]. The highest rate of *de novo* synthesis of these FAs occurs in liver and adipose tissue, which converts the excess of glucose into FAs for storage and transport [4]. During *de novo* synthesis of FAs, palmitic acid (C16:0) produced by cytoplasmic acetyl-CoA carboxylase (ACC) and fatty acid synthase (FASN) is transferred to endoplasmic reticulum membranes, where FA elongase and desaturase enzymes catalyze the conversion of saturated FAs into monounsaturated FAs, such as palmitoleic acid (C16:1(n-7)) or oleic acid (C18:1(n-9)) [5], [6]. Accordingly, FA elongase activity has an important role in regulating the synthesis of *de novo*-derived MUFAs and establishing the balance among C16:1(n-7), C18:1(n-7) and C18:1(n-9) [6].

In 2003, Clop et al. identified a QTL on porcine chromosome 8 (SSC8) with significant effects on C16:0 and C16:1(n-7) contents and a suggestive effect on C18:1(n-9) detected in backfat, using an Iberian x Landrace F_2_ intercross (IBMAP) [7]. Previous studies in our group evaluated the *MTTP* gene as a positional candidate gene for this QTL fatty acid composition detected on SSC8 [8]. A mutation in the lipid transfer region of the *MTTP* protein (p.Phe840Leu) was associated with fatty acid composition of porcine fat and with the *MTTP* lipid transfer activity measured with an *in vitro* assay. Furthermore, two QTL regions in 62 and 92 cM on SSC8, related with C16:0 and C16:1(n-7) fatty acid content in *Longissimus dorsi* muscle, respectively, were detected in a Chinese cross between Duroc and Erhualian [9]. More recently, a Genome-Wide Association Study (GWAS), performed on *Longissimus dorsi* muscle fatty acid composition from an Iberian x Landrace backcross population, detected this QTL between positions 92.1 Mb-96.7 Mb on SSC8 (according to Sscrofa 9.61 genome assembly) at 10 Mb from the *MTTP* gene [10]. This QTL was also identified using backfat fatty acid composition at positions 89 cM (C16:0) and 91 cM (C16:1(n-7) (Muñoz *et al*. (2012), manuscript in preparation). In this region, a relevant gene for fatty acid metabolism has been located: *ELOVL fatty acid elongase 6* (*ELOVL6*). The *ELOVL6* gene is a member of the elongation-of-very-long-chain-fatty-acid gene family (*ELOVLs*) of condensing enzymes that perform the first and rate-limiting step in the elongation cycle in mammals [11]. These enzymes use malonyl-CoA as the 2-carbon donor to initialize the elongation process. In pigs, the family of enzymes consists of at least seven members, differing in their substrate preferences for FAs of different lengths and degrees of unsaturation, and specific spatial and temporal expression. To generalize, FA elongases can be divided into two major groups: a) enzymes involved in the elongation of saturated and monounsaturated very-long-chain fatty acids (*ELOVL1*, *3*, *6* and *7*) and b) enzymes which are elongases of polyunsaturated fatty acids (*ELOVL2*, *4* and *5*) [12], [13]. The *ELOVL fatty acid elongase 6* (*ELOVL6*) gene (also known as LCE and FACE) is the only elongase involved in *de novo* lipogenesis, which catalyzes the elongation of long-chain saturated and monounsaturated FAs with 12–16 carbons to C18, but it does not possess activity beyond C18 [11]. Analysis of *ELOVL6-*deficient mice demonstrated that *ELOVL6* plays a crucial role in the overall fatty acid composition balance [5], and alterations in this composition have important effects on *de novo* lipogenesis and fatty acid oxidation [5]. The clear relationship between *ELOVL6* function and the QTL phenotype makes this gene a promising positional and functional candidate gene for the traits analyzed.

In the present study, a refined localization of the QTL affecting C16:0 and C16:1(n-7) FA in muscle and the evaluation of the porcine *ELOVL6* gene as candidate gene for this QTL was carried out in an Iberian x Landrace backcross population. DNA sequencing, gene expression analyses and association studies were performed to evaluate the involvement of this gene in C16:0 and C16:1(n-7) FA contents. In this article, we present different evidence that supports the role of *ELOVL6* gene polymorphism in the determination of muscle fatty acid composition in pigs.

## Materials and Methods

### Animal samples

Animals used in this study belong to the IBMAP cross, a population generated by crossing three Iberian (Guadyerbas line) boars with 31 Landrace sows [14], and containing several generations and backcrosses. The *ELOVL6* sequencing and gene expression analyses were carried out in animals from a backcross (BC1_LD) generated by crossing five F1 (Iberian x Landrace) boars with 26 Landrace sows and producing 144 backcrossed animals. All animals were maintained under intensive conditions and feeding was *ad libitum* with a cereal-based commercial diet. Animal care and procedures were performed following national and institutional guidelines for the Good Experimental Practices and approved by the Ethical Committee of the Institution (IRTA- Institut de Recerca i Tecnologia Agroalimentàries). Animals were slaughtered at an average age of 179.8±2.3 days, and samples of liver, muscle (*Longissimus dorsi*) and adipose tissue (backfat) were collected, snap-frozen in liquid nitrogen and stored at −80°C until analyzed. Genomic DNA was obtained from blood samples of all animals by the phenol-chloroform method, as described elsewhere. Composition of fatty acid with 12 to 22 carbons was determined in muscle [10] and backfat (Muñoz *et al*. (2012), manuscript in preparation) using a protocol based on gas chromatography of methyl esters [15].

### Linkage map and haplotype reconstruction

A total of 439 animals, including the founder populations, were genotyped with the Porcine SNP60K BeadChip [16]. CRI-MAP version 2.503, developed by Evans and Maddox [http://www.animalgenome.org/bioinfo/tools/share/crimap], was used to build the linkage map using the genotype information of SSC8. In addition, previously detected polymorphisms in the *MTTP* and *FABP2* genes were also included in the analysis [8], [17]. Raw data had a high genotyping quality (call rate >0.99) and, after selecting SNPs with MAF >5%, markers with genotyping and mapping errors were excluded by using the “Chrompic” option of CRI-MAP and R scripts developed by our group. Finally, we recalculated the genetic distances, employing the “Fixed” option, and 2,565 SNPs were retained for subsequent analyses (Table S1). Haplotypes were reconstructed using DualPHASE software [18], which exploits population (linkage disequilibrium) and family information (Mendelian segregation and linkage) in a Hidden Markov Model setting.

### Chromosome 8 association and fine-mapping analyses

GWAS for the intramuscular profile of palmitic and palmitoleic acids was performed with a mixed model [19], [20] accounting for additive effects associated with each marker (see below) by using Qxpak 5.0 [21]:
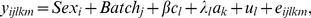
(1)in which y_ijlkm_ is the l-th individual record, sex (two levels) and batch (five levels) are fixed effects, β is a covariate coefficient with *c* being carcass weight, λ_l_ is a −1, 0, +1 indicator variable depending on the l-th individual genotype for the k-th SNP, a_k_ represents the additive effect associated with SNP, u_l_ represents the infinitesimal genetic effect treated as random and distributed as N(0, **A**σ_u_) where **A** is a numerator of the kinship matrix and e_ijlkm_ is the residual. The same model was carried out for studying the association of polymorphisms detected in the *ELOVL6* gene with palmitic and palmitoleic acid profiles in muscle and backfat.

QTL fine-mapping was performed by simultaneously exploiting linkage and linkage disequilibrium (LD) using a haplotype-based approach [18] and following the mixed model:
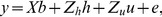
(2)in which *b* is a vector of fixed effects (sex and batch), *h* is the vector of random QTL effects corresponding to the K cluster defined by the Hidden State (HS), *u* is the vector of random individual polygenic effects and *e* is the vector of individual error. The genome-wide significance was determined using the R-package q-value [22], and the cut-off of the significant association was set at q-value≤0.05.

In order to estimate the LD between the SNPs located within the candidate region, a LD analysis was performed using the genotype and phases information from DualPHASE software. The LD estimated for each pair of SNPs was visualized using the “LDheatmap 0.9” R package [23].

### RNA isolation and cDNA synthesis

Total RNA was obtained from liver, muscle and backfat tissues using the RiboPure™ Isolation of High Quality Total RNA (Ambion®), following the manufacturer's recommendations. RNA was quantified using the NanoDrop ND-1000 spectrophotometer (NanoDrop products) and checked for purity and integrity in a Bioanalyzer-2100 (Agilent Technologies). The isolated RNA was reverse-transcribed into cDNA using the High-Capacity cDNA Reverse Transcription kit (Applied Biosystems) and random hexamers in a total volume of 20 µl containing 1 µg (liver and muscle) or 0.3 µg (backfat) of total RNA, following the manufacturer's instructions.

### Amplification and sequencing of the pig *ELOVL6* coding region and proximal promoter

The proximal promoter and the entire coding region of the *ELOVL6* gene was amplified and sequenced in twenty samples from the BC1_LD. Primers (Table S2) to amplify two overlapping fragments of 688 bp and 499 bp, including the complete coding region, were designed from the human GenBank NM_024090.2 sequence, assuming conservation across species. The proximal promoter region was amplified for the Sus scrofa breed mixed chromosome 8 sequence (GenBank:NW_003610943) available at the Sscrofa10.2 database (primers in Table S2) and assuming conservation with the human and mouse *ELOVL6* promoters [24]. A total of 1046 bp of the *ELOVL6* promoter and exon 1 were sequenced in two overlapping fragments of 604 bp and 605 bp. Primers were designed using the software PRIMER3 [25] and were validated using the software PRIMER EXPRESS™ (Applied Biosystems).

PCRs were carried out in a total volume of 25 µl containing 0.6 units of AmpliTaq Gold (Applied Biosystems), 1.5–2.5 mM MgCl_2_ (depending on the primers; see Table S2), 0.2 mM of each dNTP, 0.5 µM of each primer and 50 ng of genomic DNA or 2 µl of cDNA. Thermocycling was carried out under the following conditions: 94°C for 10 min, 35 cycles of 94°C for 1 min, 58°C–62°C (depending on the primers; see Table S2) for 1 min and 72°C for 1 min, with a final extension of 72°C for 7 min.

PCR products were purified using the ExoSAP-IT® method and sequenced with a Big Dye Terminator v.1.1 Cycle Sequencing Kit in an ABI 3730 analyzer (Applied Biosystems).

To characterize the *ELOVL6* promoter, a computer-assisted identification of putative promoter/enhancer elements was performed using the TFSEARCH software [http://www.cbrc.jp/research/db/TFSEARCH.html] and MATINSPECTOR application (set at a cut-off score of >85%) [26], a part of GENOMATIXSUITE software (Genomatix Software GmbH). Genomatix Matrix Library 8.3 was used with a core similarity threshold of 0.85 and an optimized matrix similarity threshold.

### Gene expression quantification

A total of 110 animals of the BC1_LD backcross were selected to perform gene expression quantification in liver, backfat and muscle. PCR primers were designed using PRIMER EXPRESS™ software (Applied Biosystems) and are shown in Table S2. Primers for amplification of *ELOVL6* mRNA were designed from the available sequence (GenBank:XM_003357048) covering exons 3–4 to amplify a 103-bp-long fragment. Three genes frequently used as references in RT-qPCR experiments were analyzed as endogenous controls: *β-2 microglobulin* (*β2M*), *Hypoxanthine phosphoribosyltransferase1* (*HPRT1*) and *Glyceraldehyde 3-phosphate dehydrogenase* (*GAPDH*) [27], [28]. All reference genes were tested using the software GeNorm [29], and the two best endogenous controls for all tissues were *β2M* and *HPRT1*. PCR amplification was performed in triplicate in a 20 µl final volume containing 2 µl of cDNA sample, diluted 1∶20 in DEPC-treated H_2_O from liver and muscle samples, and 1∶5 from backfat samples. For gene amplification, FastStart Universal SYBR Green Master (Rox; Roche Applied Biosystems) was used. Primers were used at 900 nM for the *ELOVL6* gene and 600 nM for both references genes, except from *HPRT1* in the muscle study (900 nM). PCR amplification was run on an ABI PRISM 7900HT Sequence Detection System (Applied Biosystems) using 96-well optical plates under the following conditions: 10 min at 95°C, 40 cycles of 15 sec at 95°C and 1 min at 60°C. A dissociation curve was drawn for each primer pair to assess that there was no primer-dimer formation.

To quantify and normalize the relative quantification (RQ) data, the 2^−ΔΔCT^ method [30] was applied using a sample with low expression as a calibrator. Comparison of mean values between genotypes was made using a linear procedure of R software, which employs a single stratum analysis of variance considering sex and batch as fixed effects. Differences were considered statistically significant at a p-value of < 0.05.

### Genotyping

BC1_LD backcross animals (N = 144) were genotyped with the Porcine SNP60 BeadChip (Illumina) using the Infinium HD Assay Ultra protocol (Illumina). Raw data had a high genotyping quality (call rate >0.99) and was visualized and analyzed with the GenomeStudio software (Illumina). For subsequent data analysis, a subset of 54,998 SNPs was selected by removing the SNPs with a minor allele frequency <5%, those with missing genotypes >5% and the duplicated SNPs in the Sscrofa 10.2 assembly.

The SNPs *ELOVL6:c.-533C>T, ELOVL6:c.-480C>T* and *ELOVL6:c.416C>T* were genotyped using the KASP SNP genotyping system platform [http://www.kbioscience.co.uk/reagents/KASP/KASP.html]. A total of 160 animals were genotyped, 125 of those belonging to BC1_LD and the rest being parental animals of the IBMAP cross (F0 and F1).

### GWAS of gene expression

Association analyses of RT-qPCR expression data of *ELOVL6* mRNA in liver, backfat and muscle, and whole-genome SNP genotypes, were carried out with Qxpak 5.0 software. The position of the SNPs was based on the *Sus scrofa* 10.2 genome assembly [http://www.animalgenome.org/repository/pig/]. For GWAS analysis, the previously described model (1), without correcting for carcass weight, was used. The infinitesimal effect allows us to adjust the data for family information and, thus, to correct the inter-chromosomal linkage disequilibrium effect. In this analysis, each SNP was tested individually to check the association. Chromosome X was analyzed using the same models, but including a dosage compensation parameter [31]. The R package q-value [22] was used to calculate the FDR-based q-value to measure the statistical significance at the genome-wide level for association studies. The cut-off of significant association at the whole genome level was set at q-value≤0.1. This significance threshold is likely too stringent due to the linkage association among SNP genotypes. Gene annotation for 2 Mb genomic intervals around the most significant SNPs was performed with *Biomart* software in the Ensembl Sscrofa 10.2 data set [www.ensembl.org]. For gene annotation, only those regions that showed a cut-off at a chromosome-wise level lower than q-value < 0.05 were selected.

## Results

### Linkage and haplotype reconstruction

The length of the linkage map on SSC8 was 131.2 cM and the ratio between the genetic and the physical distance was 0.89 cM/Mb, similar to that previously reported [32]. Genotypes from a total of 2,565 SNPs of the Porcine SNP60 BeadChip (Illumina) were employed to reconstruct the haplotypes through DualPHASE software. Previous studies showed that the estimation of the phenotypic effect of haplotype clusters is a good approximation to identify the functionally relevant ones, as well as to reduce the confidence interval for the fine mapping QTL [18], [33]. In this study, a method based on Hidden Markov Models that simultaneously phases and sorts haplotypes using linkage and LD information for haplotype reconstruction was employed. A total of twenty haplotype clusters (K = 20) were used for fine mapping, as described below.

### Fine mapping and gene annotation

**Figure 1:**
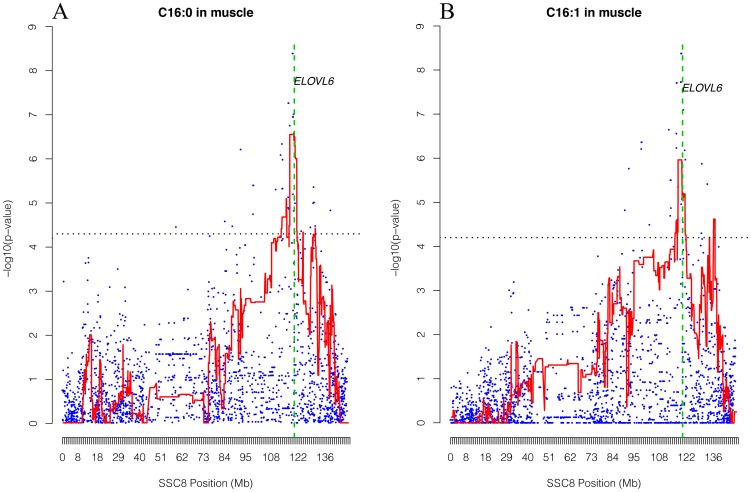
Reduction of the QTL interval by GWAS and LDLA analyses and gene mapping of *ELOVL6*. Plot of GWAS (blue points) and LDLA patterns (red line) for palmitic (A) and palmitoleic (B) acids. The X-axis represents chromosome 8 positions in Mb and the Y-axis shows the –log10 (p-value). The vertical green line represents the position of the *ELOVL6* gene on SSC8. Horizontal dashed lines mark the genome-wide significance level (FDR-based q-value≤0.05). Positions in Mb are relative to *Sscrofa10.2 assembly* of the pig genome.

A combination of the haplotype-based approach and GWAS for the intramuscular profile of palmitic and palmitoleic acids was performed in 144 BC1_LD individuals and 2,565 SNPs. It is worth noting that, for both traits, the two strategies showed the highest association at the same position (Figure 1). For instance, the GWAS profile corresponding to palmitic acid was maximized at 119,727,822–119,887,525 bp (p-value = 6.19×10^−09^) and the profile score from the haplotype-based analyses showed the maximum association signal at position 117,824,360–119,887,525 bp (p-value = 3.57×10^−07^) (Figure 1A). For palmitoleic acid, the GWAS profile was maximized at 119,851,321–120,104,023 (p-value = 4.23×10^−09^) and the profile scores from the haplotype-based analyses were maximized at position 117,824,360–119,727,822 bp (p-value = 1.09×10^−06^) (Figure 1B). In general, the association signal obtained by GWAS was higher than were curves obtained with the haplotype-based approach. However, it should be noted that the haplotype-based approach allowed us to simultaneously exploit linkage analysis and LD (LDLA). In addition, although both strategies were modeled by a mixed model, a different parameterization was employed. Thus, in the LDLA approach, HS was treated as additive random effects, whereas in GWAS a single-marker regression analysis was performed and the SNP alleles were treated as additive fixed effects.

According to the fine mapping data, the region comprised between 117–121 Mb was annotated using *Biomart* software in the Ensembl Sscrofa10.2 dataset [www.ensembl.org]. A total of 21 genes were located in this region, but only two were clearly related to fatty acid metabolism: *ELOVL6* (at position 120,119,244 bp) and *PLA2G12A* (at position 120,566,787 bp). The coincidence between the biological function of *ELOVL6* and the observed QTL effect on fatty acid composition on SSC8 strengthens the interest of the *ELOVL6* gene as the positional candidate gene for this QTL.

### Identification of polymorphisms in the porcine *ELOVL6* gene

To characterize the porcine *ELOVL6* gene, a 1,046-bp long fragment of the *ELOVL6* promoter and exon 1 was amplified from genomic DNA and sequenced, assuming conservation with the human and mouse genes. In addition, the entire coding region of the *ELOVL6* gene was amplified and sequenced. The alignment and analysis of these sequences allowed for the identification of eight polymorphisms (Table 1): one synonymous polymorphism in exon 4 and seven nucleotide substitutions in the promoter region. The SNPs located in the promoter were arranged in three haplotypes, which can be distinguished by genotyping the *ELOVL6:c.-533C>T* and *ELOVL6:c.-480C>T* polymorphisms (relative to the transcription start site, TSS, of the GenBank:NW_003610943). Hence, these two tag polymorphisms and the *ELOVL6:c.416C>T* SNP in exon 4 (GenBank:AB529461) were genotyped in parental and BC1_LD animals. Regarding the IBMAP founders, the *ELOVL6:c.-533C* allele and *ELOVL6:c.416T* allele were fixed in Iberian boars. The allele frequencies for these two SNPs were 0.25 for F1 Landrace sows and 0.78 and 0.72 for the BC1_LD Landrace sows, respectively. In contrast, *ELOVL6:c.-480C>T* SNP was not fixed in the Iberian founders, and therefore it was less informative. Both *ELOVL6:c.-533C>T* and *ELOVL6:c.416C>T* polymorphisms segregated in the BC1_LD animals with frequencies of 0.63 for allele C and 0.60 for allele T, respectively. Linkage disequilibrium analysis revealed that the three *ELOVL6* polymorphisms were in strong LD (D′ = 0.99) with three of the most significant SNPs (SIRI0000509, INRA0030422 and H3GA0025321) identified in both GWAS and fine mapping analyses (Figure S1).

**Table 1:** Polymorphisms identified in the proximal promoter and coding regions of the *ELOVL6* gene.

Gene localization	Position (bp)	Polymorphism
**Promoter** 1	−574	C/T
	−534	C/T
	−533^3^	C/T
	−492	G/A
	−480^3^	C/T
	−394	G/A
	−313	C/T
**Exon 4** 2	+416^3^	C/T

1 Positions relative to the transcription start site using, as reference, the GenBank NW_003610943 sequence.

2 Referring to the coding region (GenBank:AB529461).

3 SNPs genotyped in the BC1_LD population.

**Figure 2:**
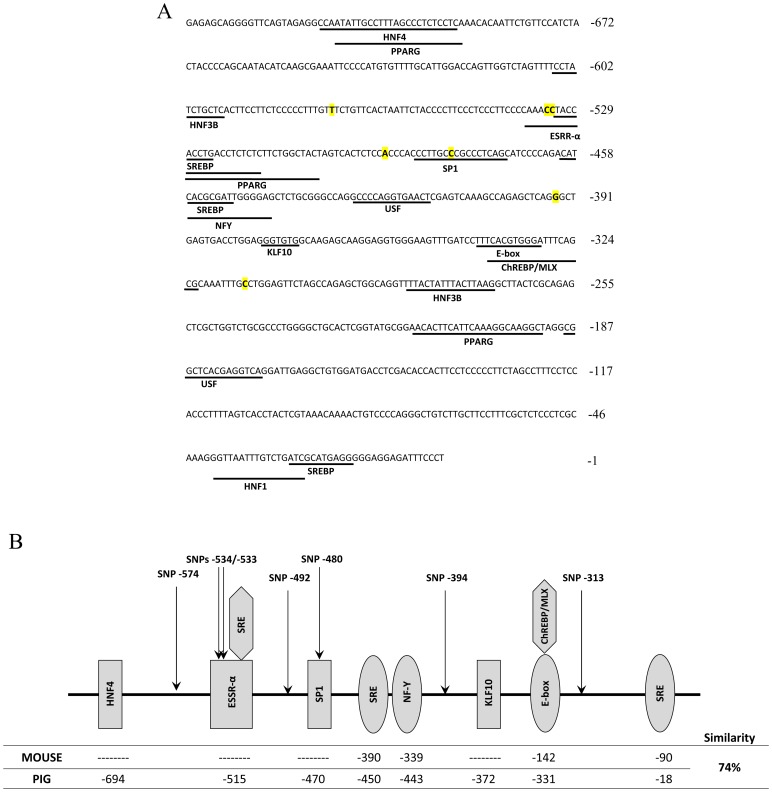
Genetic characterization of the *ELOVL6* pig promoter and identification of potential cis-acting DNA-sequence motifs. Summary of the *ELOVL6* pig promoter: A, nucleotide sequence of the 5′-flanking region of the porcine *ELOVL6* gene, where potential binding sites for transcription factors are underlined. Positions of *ELOVL6* promoter polymorphisms are labeled in yellow. B, comparison of transcription factor binding sites between mouse and the pig *ELOVL6* promoter, including *ELOVL6* SNPs localization.

To assess if polymorphisms in the promoter region could affect *ELOVL6* expression through the disruption of transcription factor-binding sites, a computer-assisted identification of potential cis-acting DNA-sequence motifs was carried out. As has been previously described in mouse liver, the *ELOVL6* gene is regulated by SREBP-1 [4], [24], [34], [35]. SREBP-1 presents dual DNA sequence specificity, binding to both E-box and SRE motifs [36]. Four SREBP binding sites were identified in the pig *ELOVL6* promoter, three SRE elements in positions −27 to −17, −460 to −449 and −532 to −524 (Figure 2) and one E-box in position −341 to −330 (Figure 2A), relative to the TSS of the GenBank sequence NW_003610943, similar to those observed in the mouse promoter (Figure 2B) [24]. Also, other candidate transcription factors, with biological relevance, have elements in this promoter, such as MLX (at position −339 to −322), which belongs to the family of basic helix-loop-helix leucine zipper (bHLH-Zip) and induces *ELOVL6* gene expression by glucose in mice [6], HNF4γ (at position −719 to −694) or KLF10 (at position −377 to −372) (Figure 2). However, none of our polymorphisms changed these binding sites. Interestingly, two consecutive SNPs forming a haplotype at positions −533 (*ELOVL6:c.-533C>T)* and *−534 (ELOVL6:c.-534C>T)* were identified in the core binding site of the *estrogen-related receptor alpha* gene (*ESRR-α*), generating a multi-nucleotide polymorphism. Furthermore, the *ELOVL6:c.-480C>T* polymorphism was also located in a potential SP1 binding site (Figure 2).

### Association of *ELOVL6* polymorphisms with C16:0 and C16:1(n-7) composition in muscle and backfat

An association analysis with the SSC8 genotypes from 2,565 SNPs of the Porcine SNP60 BeadChip (Illumina) and three *ELOVL6* SNPs in 125 BC1-LD animals was performed using the additive model (1). In this analysis, the *ELOVL6:c.-533C>T* polymorphism showed the highest association with the percentage of palmitic acid (p-value = 1.38×10^−07^; â(estimated additive effect) = 0.742; see Figure S2A) and palmitoleic acid (p-value = 1.23×10^−08^; â = 0.253; see Figure S2B) content in muscle. Also, a relevant association was observed between *ELOVL6:c.416C>T* polymorphism with palmitic, and palmitoleic acid content in muscle (p-value (C16:0) = 1.11×10^−04^; â(C16:0) = 0530; p-value (C16:1(n-7)) = 6.98×10^−07^; â(C16:1(n-7)) = 0.214; see Figure S2). In backfat, the *ELOVL6:c.-533C>T* polymorphism was the most significantly associated one with palmitic acid content (p-value = 2×10^−15^; â = 0.976). In addition, *ELOVL6:c.416C>T* SNP showed a high association with palmitic acid content (p-value = 6.27×10^−13^; â = 0.859) (data not shown). Analyzing the palmitoleic acid content in backfat, the most significantly associated SNP was H3GA0025290 (113,528,768 bp; â = 0.182, p-value = 8.54×10^−10^). *ELOVL6* polymorphisms *ELOVL6:c.-533C>T* and *ELOVL6:c.416C>T* also showed a significant association with palmitoleic content: p-value = 6.14×10^−09^ (â = 0.168) and p-value = 6.95×10^−08^ (â = 0.151), respectively (data not shown). The clear association of the *ELOVL6:c.-533C>T* polymorphism with the percentage of both fatty acids in muscle and backfat yields new evidence to continue studying *ELOVL6* as a candidate gene for SSC8 QTL.

### Effect of the *ELOVL6: c.-533C>T* polymorphism on gene expression and fatty acid composition

The association of the *ELOVL6: c.-533C>T* polymorphism with the percentages of C16:0 and C16:1(n-7) suggests a role of this mutation in the regulation of *ELOVL6* gene expression and, subsequently, in fatty acid metabolism. Thus, the expression profile of the pig *ELOVL6* gene, in liver, backfat and muscle, organs particularly important in fatty acid metabolism, was studied by RT-qPCR in 110 BC1_LD animals. In accordance with previous results in mouse and rat, in which high *ELOVL6* expression was found in tissues with active lipogenesis [4], [35], [37], the highest expression was found in backfat tissue, followed by liver and muscle. Clear differences in *ELOVL6* expression were observed among samples in all tissues, with a highly significant effect of sex in liver (p-value = 6.5×10^−03^), backfat (p-value = 3.4×10^−04^) and muscle (p-value = 3.4×10^−05^), where *ELOVL6* gene expression was higher in females than in males. In addition, the correlation between the *ELOVL6* expression levels across the three tissues was analyzed, but no clear associations were observed among tissues. This result suggests that the mechanisms controlling *ELOLV6* expression are different in backfat, liver and muscle tissues [35].

**Figure 3:**
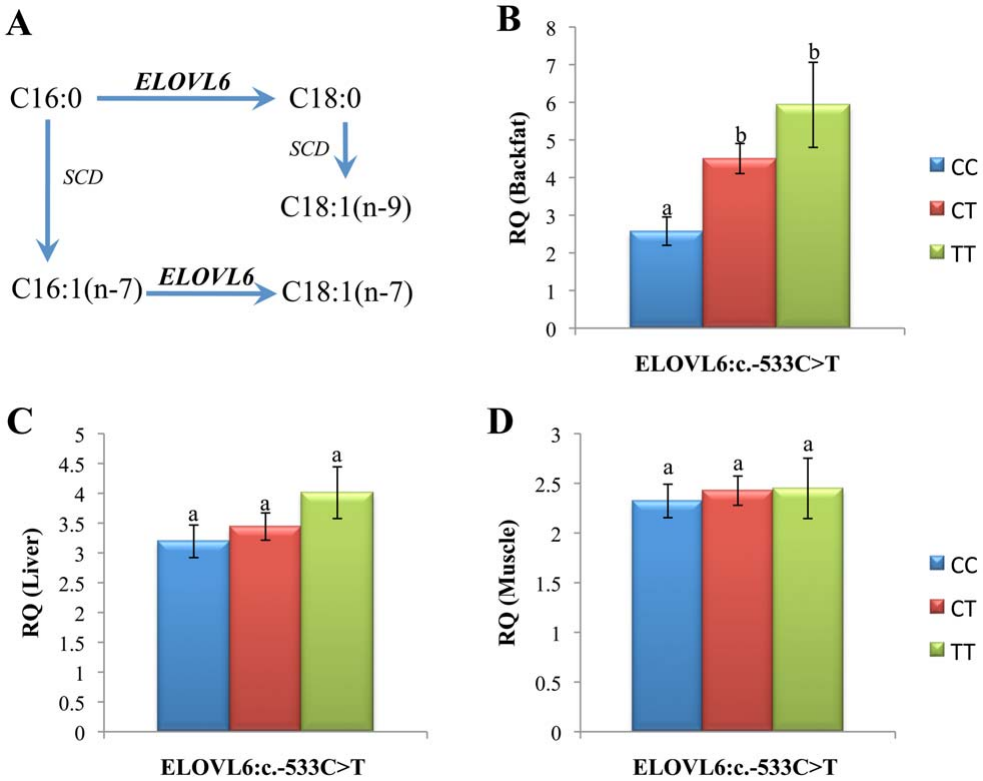
Association of *ELOVL6: c.-533C>T* genotypes on gene expression in backfat. A SNP genome-wide association study was performed with *ELOVL6* relative expression levels measured by RT-qPCR in 110 samples from backfat, liver and muscle. Data include: Schematic representation of the elongation pathway of 16-carbon fatty acid (A), *ELOVL6* expression levels in backfat (B), liver (C) and muscle (D). Data represent means ± SEM. Values with different superscript letters (a, b and c) indicate significant differences between groups (p-value < 0.05), as determined by a single stratum analysis of variance considering sex and batch as fixed effects.

**Figure 4:**
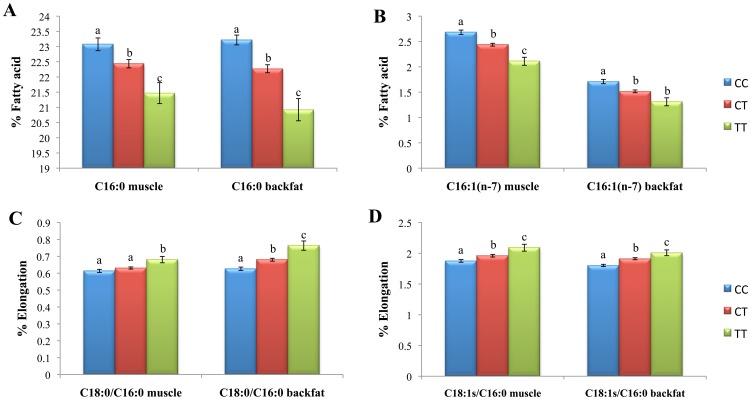
Fatty acid composition of different *ELOVL6:c.-533C>T* genotypes in muscle and backfat. Data include: percentage of C16:0 (A) and C16:1(n-7) fatty acids (B) in muscle and backfat; and the elongation ratios C18:0/C16:0 (C) and C18:1(n-7)+C18:1(n-9)/C16:0 (D) in muscle and backfat. Data represent mean ± SEM. Values with different superscript letters (a, b and c) indicate significant differences between groups (p-value < 0.05), as determined by a single stratum analysis of variance considering sex and batch as fixed effects.

When animals were classified according to the *ELOVL6:c.-533C>T* genotypes, no significant variations were found between genotypes when liver and muscle samples were analyzed (Figure 3C–D). Nevertheless, different levels of expression between genotypes were obtained in backfat samples (p-value = 8.7×10^−05^; Figure 3B), where animals with the CC genotype showed a significantly lower expression, as compared to animals with the other two genotypes. Interestingly, when only individuals with a known allele origin for the *ELOVL6:c.-533C>T* polymorphism were analyzed, the Iberian allele C decreased the expression, in comparison with the Landrace allele T (p-value = 4.6×10^−03^). Accordingly, CC homozygous individuals showed a higher percentage of C16:0 in muscle (p-value = 3.61×10^−05^) and backfat (p-value = 1.83×10^−09^), in comparison with TT individuals (Figure 4A). Similar results were obtained for C16:1(n-7) in both tissues, with CC animals presenting a higher relative content of this fatty acid (p-value (muscle) = 7.1×10^−06^ and p-value (backfat) = 1.47×10^−04^; Figure 4B). These data suggest a substrate accumulation in individuals with the C allele due to a hypothetical deficiency of the *ELOVL6* gene (Figure 3A). In agreement with these data, a decrease of C18:0 content was also observed in backfat (p-value = 5×10^−02^) in animals with the C allele, but such differences were not present in muscle (data not shown). Although non-significant differences were observed in the 18-carbon fatty acid content in muscle and backfat (except for the C18:0 in backfat), a significant decrease in elongation activity ratios (C18:0/C16:0 and C18:1(n-7)+C18:1(n-9)/C16:1(n-7)) were observed in both tissues in animals with the CC genotype (Figure 4C–D).

### Genome-wide association studies of *ELOVL6* gene expression

**Figure 5:**
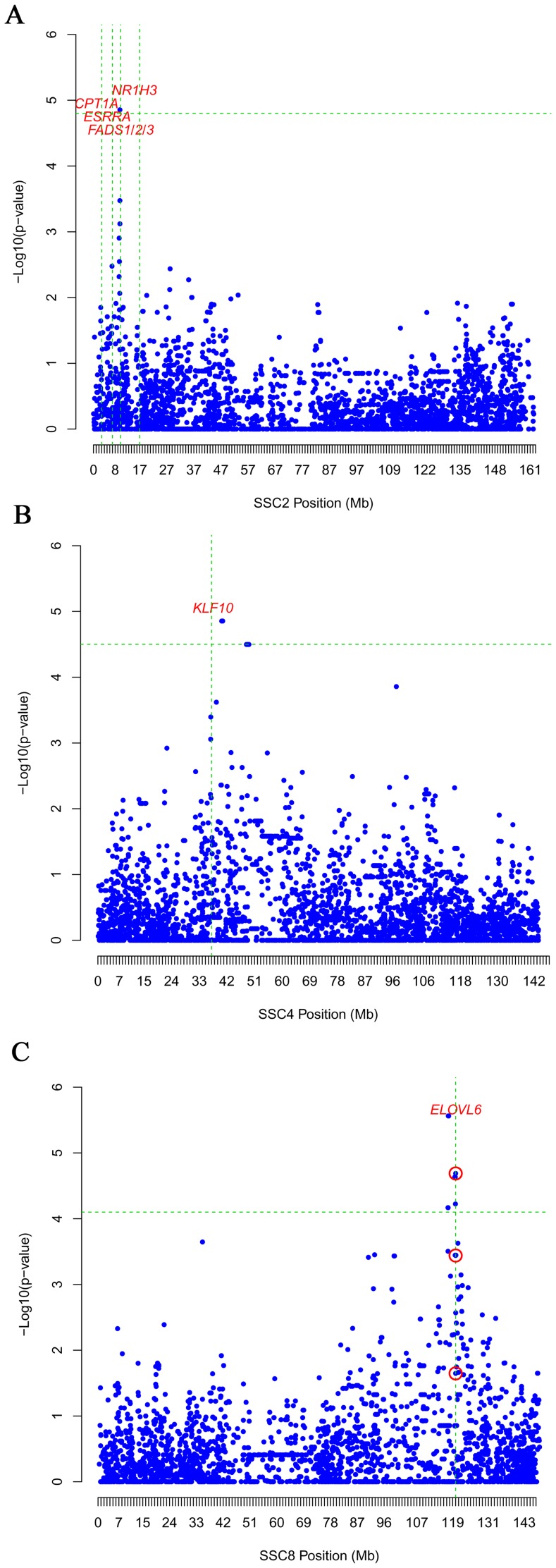
Significant region obtained in GWAS for backfat gene expression. Association analysis between the backfat *ELOVL6* expression level and SNP genotypes for SSC2 (A), SSC4 (B) and SSC8 (C). *ELOVL6* polymorphisms are included and labeled with a red circle. Positions in Mb are relative to *Sscrofa10.2 assembly* of the pig genome. Vertical, dashed lines indicate the location of positional candidate genes. Horizontal, dashed lines mark the genome-wide significance level (FDR-based q-value≤0.1).

**Figure 6:**
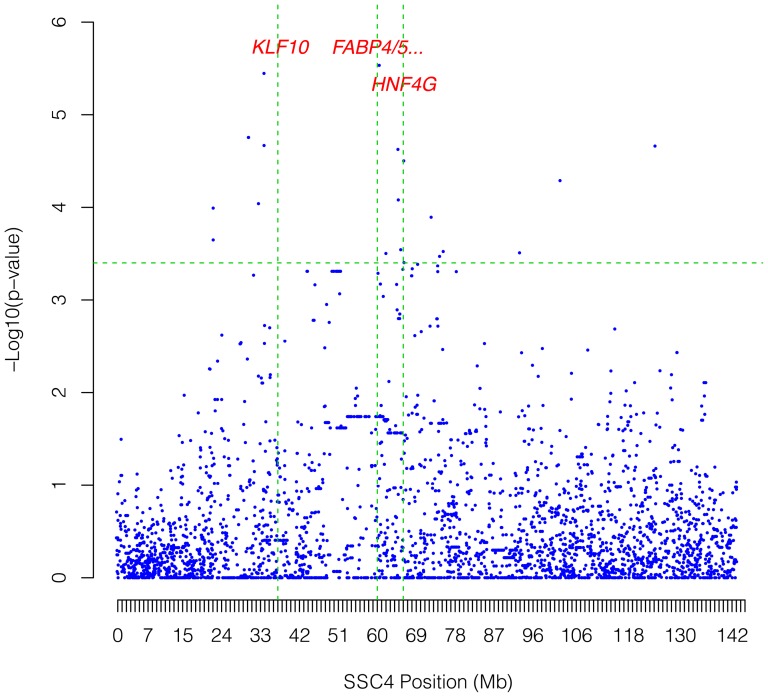
Significant region obtained in GWAS for liver gene expression. Association analysis between the liver *ELOVL6* expression level and SNP genotypes for SSC4. Positions in Mb are relative to *Sscrofa10.2 assembly* of the pig genome. Vertical, dashed lines indicate the location of positional candidate genes. Horizontal, dashed lines mark the chromosome-wide significance level (FDR-based q-value≤0.1).

Taking into account that differences in *ELOVL6* gene expression were observed among tissues and animals, GWASs using the RQ expression data of the three tissues and the genotypes of 54,998 SNPs distributed across the pig genome were carried out to find new, potential genomic regions associated with *ELOVL6* gene expression. The promoter *ELOVL6* SNPs (*ELOVL6:c.-533C>T* and *ELOVL6:c.-480C>*) and the protein-coding region SNP (*ELOVL6:c.416C>T*) genotyped in this work, which are not included in the porcine SNP60 Bead-Chip, were incorporated into the study. First, backfat analysis of *ELOVL6* gene expression showed three relevant regions in chromosomes SSC2, SSC4 and SSC8 which were significant at a chromosome-wise level (Figure S3A). Interestingly, the most significant peak was localized in SSC8 inside the QTL region, very close to the *ELOVL6* gene (ALGA0049135; 117,548,144 bp; p-value = 2.74×10^−06^) (Figure 5C). High association was also obtained with the *ELOVL6:c.-533C>T* polymorphism (p-value = 2.05×10^−05^), whereas the other two *ELOVL6* polymorphisms were not significantly associated. Gene annotation of the other two regions was performed to find potential trans-acting genetic variants modulating *ELOVL6* gene expression. In SSC2, a significant region was found between positions 9.3 Mb and 9.8 Mb (DIAS0000337; 9,736,754 bp; p-value = 1.4×10^−05^), in which several genes related to lipid metabolism were identified (Figure 5A). The most interesting ones were the *estrogen-related receptor alpha* (*ESRRα*), three genes which are members of the *fatty acid desaturase* family (*FADS1, FADS2* and *FADS3*), the *carnitine palmitoyltransferase 1A* (*CPT1A*) and the *nuclear receptor subfamily 1, group H, member 3* (*NR1H3*). Finally, the most significant region of SSC4 was found between positions 36 Mb and 44 Mb (ASGA0088888; 40,318,092 bp; p-value = 1.4×10^−05^), where the *Kruppel-like factor 10* (*KLF10*) gene was annotated (Figure 5B). In liver, three candidate chromosomal regions were significantly associated with *ELOVL6* gene expression at a chromosomal level on SSC4, SSC5 and SSC9 (Figure S3B). The most significant region in SSC4 showed two peaks at the 30 Mb–35 Mb and 60 Mb–67 Mb regions (Figure 6). Gene annotation of both regions allowed us to identify several genes, which may be related to *ELOVL6* RQ, near the two most significant SNPs: ALGA0025162 (60,844,160 bp; p-value = 2.93×10^−06^) and ALGA0024413 (34,206,333 bp; p-value = 3.58×10^−06^). Proximal to ALGA0025162 was located the *hepatocyte nuclear factor 4 gamma* (*HNF4γ*) and members 4 and 5 of the *fatty acid binding protein* family (*FABP4* and *FABP5*) (Figure 6). SNP ALGA0024413 was near the significant region detected in backfat analysis, suggesting a co-effect in both tissues by the porcine *KLF10* gene (Figure 6). No relevant genes were found in the 71 Mb to 79 Mb region of SSC5. In SSC9, the significant region was located in the 65 Mb–71 Mb interval, in which the *acyl-CoA dehydrogenase 8* (*ACAD8*) was located. Finally, muscle *ELOVL6* gene expression was asociated with three regions in SSC6, SSC8 and SSC12 (Figure S3C). The most significant one was situated in SSC6 between positions 18 Mb and 26 Mb (ALGA0034806; 19,862,636 bp; p-value = 4.02×10^−06^), where the general transcription factor *SET domain containing 6* (*SETD6*) was identified (data not shown). In SSC8 and SSC12, a significant region was found in intervals 15 Mb–19 Mb and 10 Mb–14 Mb, respectively (data not shown). Nevertheless, no relevant genes were identified using the current porcine gene annotation information. The significance threshold was likely too stringent owing to the linkage dependence among the SNPs included in the analysis and, thus, other suggestive SNP peaks may also contain relevant genes.

## Discussion

The QTL affecting palmitic and palmitoleic acid contents on SSC8 was previously identified and the porcine *MTTP* gene was analyzed as a positional candidate gene [8]. These studies were performed using a reduced number of microsatellite markers and, as a consequence, the confidence interval had several Mb. The improvements in the porcine genome and the use of the SNP data from the Illumina 60 K porcine chip allowed us to make a better estimation of the QTL position by GWAS and haplotype-based approaches. GWAS studies maximized the QTL peak at 10 Mb from the *MTTP* gene, in the region where the *ELOVL6* gene was located. Although the *ELOVL6* gene has been selected as a new functional and positional candidate gene, a lower effect of the *MTTP* gene cannot be ruled out.

Despite the crucial role of genes such as *ELOVL6* and members of the *SCD* family in determining the balance among C16:0, C16:1(n-7), C18:1(n-7) and C18:1(n-9) [5], [6], [38], the information regarding these genes in pigs is sparse. In this study, we characterized the porcine *ELOVL6* gene and we presented several pieces of evidence confirming that this lipogenic enzyme is highly associated with fatty acid composition in pigs. Among the eight polymorphisms found in the porcine *ELOVL6* gene, the *ELOVL6:c.-533C>T* polymorphism was clearly associated with C16:0 and C16:1(n-7) composition in muscle and backfat. An increase of C16s' fatty acid percentage in animals with the C allele, in comparison with animals carrying only the T alleles, was observed. In accordance with the function of *ELOVL6* (Figure 3A), which elongates C16 to C18 fatty acids [5], a lower *ELOVL6* gene expression was found in the backfat of animals with the Iberian allele. The lower *ELOVL6* gene expression was associated with the accumulation of C16:0 and C16:1(n-7) in muscle and backfat, as has been previously described in mammalian cells by modulating *ELOVL6* activity with siRNA [5], [6]. Similar results were obtained using mice deficient for *ELOVL6* [5], where an increase of C16 fatty acids and a decrease of C18 fatty acids was observed in *ELOVL6* ^−/−^ mice. In agreement with these studies, the percentage of C18:0 showed a decrease in backfat, but no differences were observed in C18:1(n-7).

The relevance of adipose tissue in overall fatty acid synthesis in pigs must be considered for the interpretation of the present results. Liver and adipose tissue are the principal organs implicated in *de novo* lipogenesis, although their contribution differs across species. In ruminants, such as cow and sheep, both liver and adipose tissue appear to be important sites of synthesis [39], while in mouse and rat adipose tissue accounts for at least 50% of the newly synthesized fatty acids [40]. Pig adipose tissue seems to be responsible for a greater contribution to overall fatty acid synthesis than does liver [41], as has been similarly observed in humans [42], [43]. In agreement with this, the expression of *ELOVL6*, a gene involved in *de novo* lipogenesis, was higher in adipose tissue than in the liver and muscle of 110 BC1_LD animals. Furthermore, the effect of SNP *ELOVL6:c.-533C>T* in *ELOVL6* expression was only significant in adipose tissue, suggesting that this polymorphism may have an influence in adipose fatty acid synthesis and, subsequently, in body fatty acid composition. In fact, adipose tissue is the major source of circulating free fatty acids (FFAs) and, together with the liver, supplies fatty acids to muscle [44]. In mice, the concentration of muscle palmitoleate is a direct reflection of adipose FFAs [44]. As in pig the contribution of adipose tissue in fatty acid synthesis is higher than in liver, we could hypothesize that the composition of fatty acids in muscle closely resembles that observed in adipose tissue [45], [46]. High and moderate positive phenotypic correlations between backfat and muscle were found for C16 and C16:1(n-7) composition (r_C16:0_ = 0.72, p-value = 2.2×10^−16^ and r_C16:1(n-7)_ = 0.43, p-value = 5.13×10^−06^, respectively), supporting our hypothesis. Furthermore, a high correlation for palmitoleic fatty acid was not expected because another genomic region with a strong effect on this fatty acid in muscle, but not in backfat, was identified in SSC4 [10].

Despite the strong association (p-value = 2.05×10^−05^) between the *ELOVL6:c.-533C>T* polymorphism and backfat *ELOVL6* gene expression, SSC8 SNP ALGA0049135 (117,548,144 bp) was more significantly associated (p-value = 2.74×10^−06^). However, *ELOVL6:c.-533C>T* showed a higher additive effect (â = 0.174), in comparison with SNPs ALGA0049135 (â = 0.154). Hence, further investigation is required to validate the *ELOVL6:c.-533C>T* polymorphism as the causal mutation or to identify new genetic variants in this QTL region modulating *ELOVL6* gene transcription that could better explain the QTL underlying phenotypic variation in C16 and C16:7(n-1).

Apart from the significant effect of SSC8 in *ELOVL6* gene expression, other interesting genomic regions were identified as being directly associated with *ELOVL6* relative expression levels in backfat, muscle and liver. Among them, a common peak at 60 Mb of SSC4 was identified for backfat and liver, suggesting the presence of genes related to *ELOVL6* expression in both tissues. The porcine *KLF10* gene was identified in this chromosomal position. It is a circadian-clock-controlled transcription factor that regulates genes involved in glucose and lipid metabolism in liver, such as *SREBP* and *ELOVL6* [47]. The identification of a potential cis-acting DNA-sequence motif for *KLF10* in the proximal promoter region of porcine *ELOVL6* supports the involvement of this gene in the *ELOVL6* transcriptional regulation in both tissues. Another interesting region associated with *ELOVL6* expression in backfat was observed in SSC2, in which the *ESRR-α* gene was identified. *ESRR-α* codes for a transcriptional regulator which binds to an ERR-α response element (ERRE) containing a single-consensus half-site 5′-TNAAGGTCA-3′ and regulates a variety of genes related to fatty acid metabolism [48]. Interestingly, this transcription factor is regulated by estrogens, the primary female sex hormones. Thus, the higher *ELOVL6* gene expression observed in females may be explained by the increase of *ESRR-α* activity, due to the high levels of estrogens in females. Furthermore, ERRE was present in the *ELOVL6* promoter and included two polymorphic positions in the core binding element (Figure 2), one of which was *ELOVL6:c.-533C>T* SNP, reinforcing this polymorphism as a candidate mutation to explain the differences in *ELOVL6* mRNA observed among animals. Additionally, the *ESRR-α* binding site overlapped one SRE motif (−532 to −524 bp), suggesting that the two polymorphisms identified in this region (*ELOVL6:c.-533C>T* and *ELOVL6:c.-534C>T*) may have an important role in selecting which transcription factor (*ESRR-α* or *SREBP1*) binds to its corresponding element. Further studies are needed to determine both the effect of the two polymorphisms to *ESRR-α* or *SREBP1* binding and how the selection of the transcription factor can affect *ELOVL6* gene expression. In liver, a second significant peak was also obtained in SSC4, in which the *HNF4γ*, *FABP4* and *FABP5* genes were identified. The porcine *HNF4γ* gene is a member of the hepatocyte nuclear receptor superfamily, which is highly homologous to *HNF4α*, suggesting that it may have a similar function in the regulation of hepatic genes [49]. The protein structure of *HNF4γ* revealed that fatty acids bind to its ligand binding pocket, acting as a regulatory molecule of *HNF4γ* [50]. In spite of the presence of the *HNF4γ* binding site in the *ELOVL6* promoter, the main relationship described between both genes is that deficiencies in *ELOVL6* gene expression deplete the newly synthesized fatty acids, which are coactivators of the *HNF4γ* gene, producing a decrease in *HNF4γ* activity [5]. Interestingly, preliminary results in our lab indicate higher expression levels of *HNF4γ* in liver than in backfat (data not shown). These data point towards *HNF4γ* as a regulator of *ELOVL6* gene expression in liver and suggest that the polymorphism proximal to or within the *HNF4γ* gene partially determines the differences in liver *ELOVL6* gene expression. On the other hand, *FABP5* is a protein that binds and transports long-chain fatty acids into the nucleus [51], where they can act as transcription factors on lipogenic genes, such as elongases [44]. Association analysis with muscle *ELOVL6* gene expression data allowed us to identify significant regions, but only general transcription factors were found. Data obtained suggest a minimal elongation activity in muscle, and probably the difference in mRNA levels between animals was caused by the intramuscular adipocytes, as was observed in previous pig studies [52]. Apart from the potential relevance of all of these genes located in significant regions in regulating *ELOVL6* gene expression, we cannot discard the involvement of other genes located in non-significant regions but with biological relevance in the regulation of *ELOVL6* expression, such as *SP1* and *SREBP* genes. In the present study, we have identified four SREBP binding sites in the pig *ELOVL6* promoter, but none of these cis-acting motifs was affected by *ELOVL6* polymorphisms. However, *SREBP* has been described as a weak transcriptional activator that requires interaction with additional regulators like NF-Y and SP1 to activate the transcription of genes involved in fatty acid metabolism [4], [53]. Interestingly, a SNP disrupting a potential SP1 binding site has been identified in the *ELOVL6* promoter, not discarding the involvement of this mutation in the differences of gene expression observed among tissues. Taking into account the regulatory networks necessary for transcriptional activation, further investigation is required to determine the role of these mutations in the *ELOVL6* expression together with the implication of tissue-specific factors and epigenetic modifications.

Finally, the results provided in the present study are both helpful for the understanding of molecular mechanisms governing important economical traits like meat quality, but also to improve the knowledge of human diseases related to obesity, including diabetes and metabolic syndrome. Fatty acid composition has been highly associated with insulin sensitivity, especially the ratio of C18 to C16 fatty acids, which is controlled by *ELOVL6* activity [5]. The accumulation of C16 fatty acids, observed in our study, has been related to protection against hepatic lipotoxicity and insulin resistance [5]. Palmitoleic acid, segregated by adipose tissue, greatly strengthens the insulin-signaling pathway, avoiding tissue insulin resistance and obesity-related diseases [44].

## Conclusions

In this work, the interval for the C16:0 and C16:1(n-7) QTL in SSC8 has been reduced, allowing for the identification of *ELOVL6* as a positional candidate gene. The characterization of the coding and proximal promoter regions of the porcine *ELOVL6* gene allowed for the identification of several mutations, especially the *ELOVL6:c.-533C>T* polymorphism strongly associated with muscle and backfat percentages of palmitic and palmitoleic acids. Interestingly, this SNP was also related to *ELOVL6* expression levels in backfat and fatty acid content and elongation activity ratios in muscle and backfat. Thus, the *ELOVL6:c.-533C>T* polymorphism is a candidate causal mutation to explain the variation in palmitic and palmitoleic acid content observed in an Iberian x Landrace cross. Hence, this work provides the first report of the importance of the porcine *ELOVL6* gene in the metabolism of fatty acids and, subsequently, in meat quality traits in pigs, but further functional studies in model organisms and validation in independent pig populations are required to confirm this causal mutation.

## Supporting Information

Table S1 **List of SNPs for SSC8 linkage map and haplotype reconstruction.**
(XLSX)

Table S2 **Primers for** ***ELOVL6*** **mRNA sequencing (R), promoter sequencing (P) and RT-qPCR (RT) study.**
(DOC)

Figure S1 **Linkage disequilibrium among** ***ELOVL6*** **polymorphisms.** Pattern of linkage disequilibrium analysis between the three identified polymorphisms on the *ELOVL6* gene and the most significant SNP detected in both GWAS and fine mapping. Figure colored from blue to red according to LD strength between consecutive markers.
(TIF)

Figure S2 **Association of SNPs from SSC8 and** ***ELOVL6*** **polymorphims with palmitic and palmitoleic acid content.** Association analyses of C16:0 (A) and C16:1(n-7) (B) with genotypes of markers included in the Porcine SNP60 Bead-Chip (Illumina). *ELOVL6* polymorphisms are included and labeled with a red circle. Positions in Mb are relative to the *Sscrofa10.2 assembly* of the pig genome. The horizontal, dashed line indicates the genome-wide significance level (FDR-based q-value≤0.05).
(TIF)

Figure S3 **GWAS for** ***ELOVL6*** **gene expression in backfat, liver and muscle.** Association analyses of *ELOVL6* expression levels in backfat (A), liver (B) and muscle (C) with genotypes of markers included in the Porcine SNP60 Bead-Chip (Illumina). Positions in Mb are relative to the *Sscrofa10.2 assembly* of the pig genome. The horizontal, dashed line indicates the genome-wide significance level (FDR-based q-value≤0.1).
(TIF)
