# Supplementary material for: Polymorphism in the ELOVL6 Gene Is Associated with a Major QTL Effect on Fatty Acid Composition in Pigs
Source: PLoS One. 2013 Jan 14;8(1):e53687. doi: 10.1371/journal.pone.0053687 (PMC3544903; doi:10.1371/journal.pone.0053687)
Supplement: Table S2 — Primers for ELOVL6 mRNA sequencing (R), promoter sequencing (P) and RT-qPCR (RT) study. (DOC) [file pone.0053687.s002.doc]

| **Name** | **Sequence** | **Amplicon lenght (bp)** | **Tm** | **[MgCl_2_]** |
| --- | --- | --- | --- | --- |
| ELOVL6-Fw1 (R) | 5'-GGAAGCAGACAGGAGAACACTC-3' | 688 | 58ºC | 2mM |
| ELOVL6-Rv1 (R) | 5'-TGATGTGGTGATACCAGTGCAG-3' |
| ELOVL6-Fw2 (R) | 5'-TCACTGTGCTCCTGTACTCTTGG-3' | 499 | 62ºC | 2.5mM |
| ELOVL6-Rv2 (R) | 5'-TAAGCTGCCTTGGGTTTTGTG-3' |
| ELOVL6-P-Fw1 (P) | 5'-GAGAGCAGGGGTTCAGTAGAGG-3' | 604 | 62ºC | 2mM |
| ELOVL6-P-Rv1 (P) | 5'-AGGAAGTGGTGTCGAGGTCATC-3' |
| ELOVL6-P-Fw2 (P) | 5'-CCAGAGCTGGCAGGTTTTACTA-3' | 605 | 62ºC | 2mM |
| ELOVL6-P-Rv2 (P) | 5'-CGGAGTCGCTACGTGTTCTCTA-3' |
| ELOVL6-RT-Fw (RT) | 5'- AGCAGTTCAACGAGAACGAAGCC -3' | 103 | 60ºC | 5mM |
| ELOVL6-RT-Rv (RT) | 5'- TGCCGACCGCCAAAGATAAAG -3' |
| HPRT1-RT-Fw (RT) | 5'-TCATTATGCCGAGGATTTGGA-3' | 91 | 60ºC | 5mM |
| HPRT1-RT-Rv (RT) | 5'-CTCTTTCATCACATCTCGAGCAA-3' |
| β2M-RT-Fw (RT) | 5'-ACCTTCTGGTCCACACTGAGTTC-3' | 100 | 60ºC | 5mM |
| β2M -RT-Rv (RT) | 5'-GGTCTCGATCCCACTTAACTATCTTG-3' |
