# Supplementary figures and images for: Polymorphism in the ELOVL6 Gene Is Associated with a Major QTL Effect on Fatty Acid Composition in Pigs
Source: PLoS One. 2013 Jan 14;8(1):e53687. doi: 10.1371/journal.pone.0053687 (PMC3544903; doi:10.1371/journal.pone.0053687)

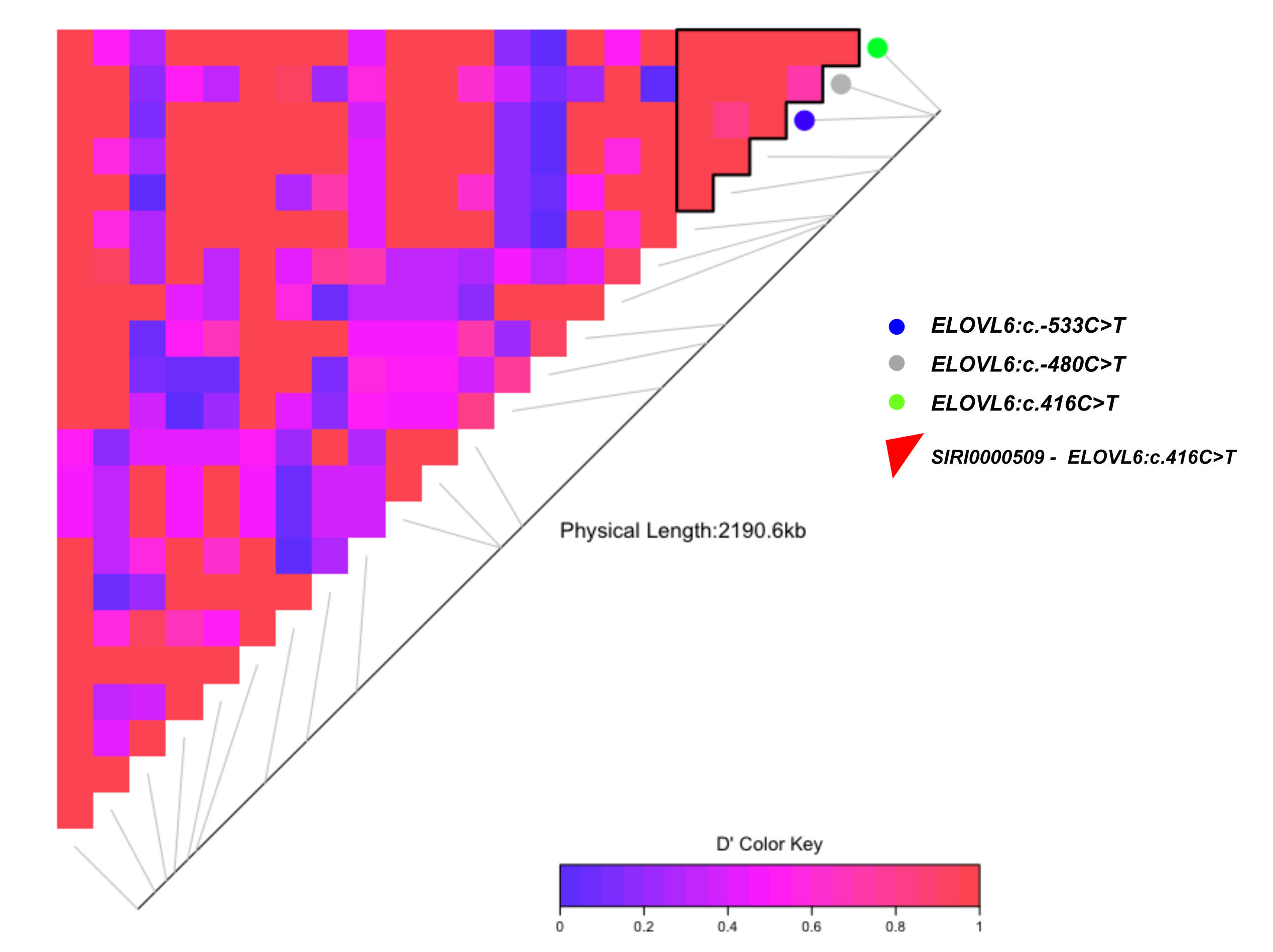

Supplement: Figure S1 — Linkage disequilibrium among ELOVL6 polymorphisms. Pattern of linkage disequilibrium analysis between the three identified polymorphisms on the ELOVL6 gene and the most significant SNP detected in both GWAS and fine mapping. Figure colored from blue to red according to LD strength between consecutive markers. (TIF) [file pone.0053687.s003.tif]

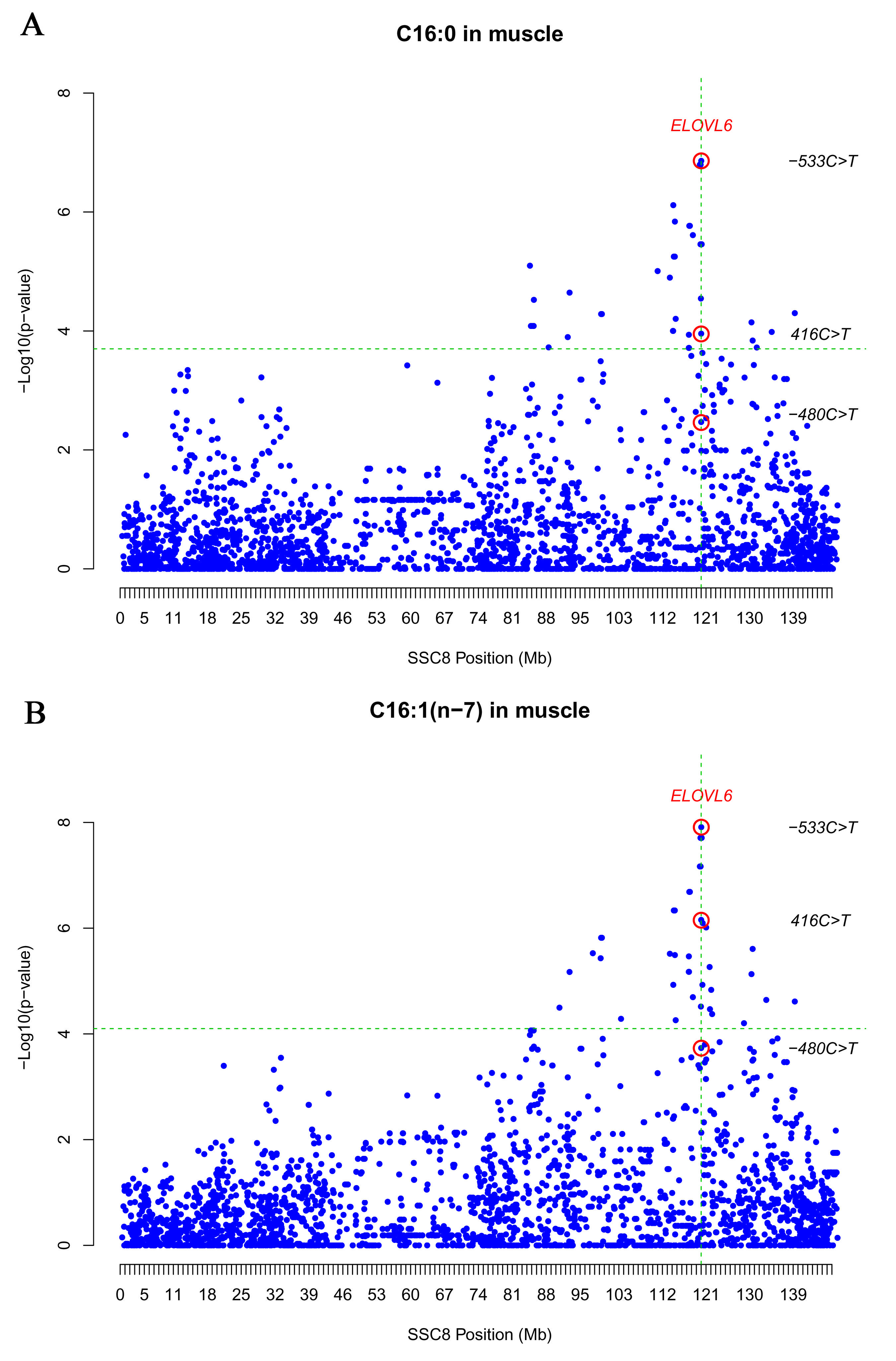

Supplement: Figure S2 — Association of SNPs from SSC8 and ELOVL6 polymorphims with palmitic and palmitoleic acid content. Association analyses of C16:0 (A) and C16:1(n-7) (B) with genotypes of markers included in the Porcine SNP60 Bead-Chip (Illumina). ELOVL6 polymorphisms are included and labeled with a red circle. Positions in Mb are relative to the Sscrofa10.2 assembly of the pig genome. The horizontal, dashed line indicates the genome-wide significance level (FDR-based q-value≤0.05). (TIF) [file pone.0053687.s004.tif]

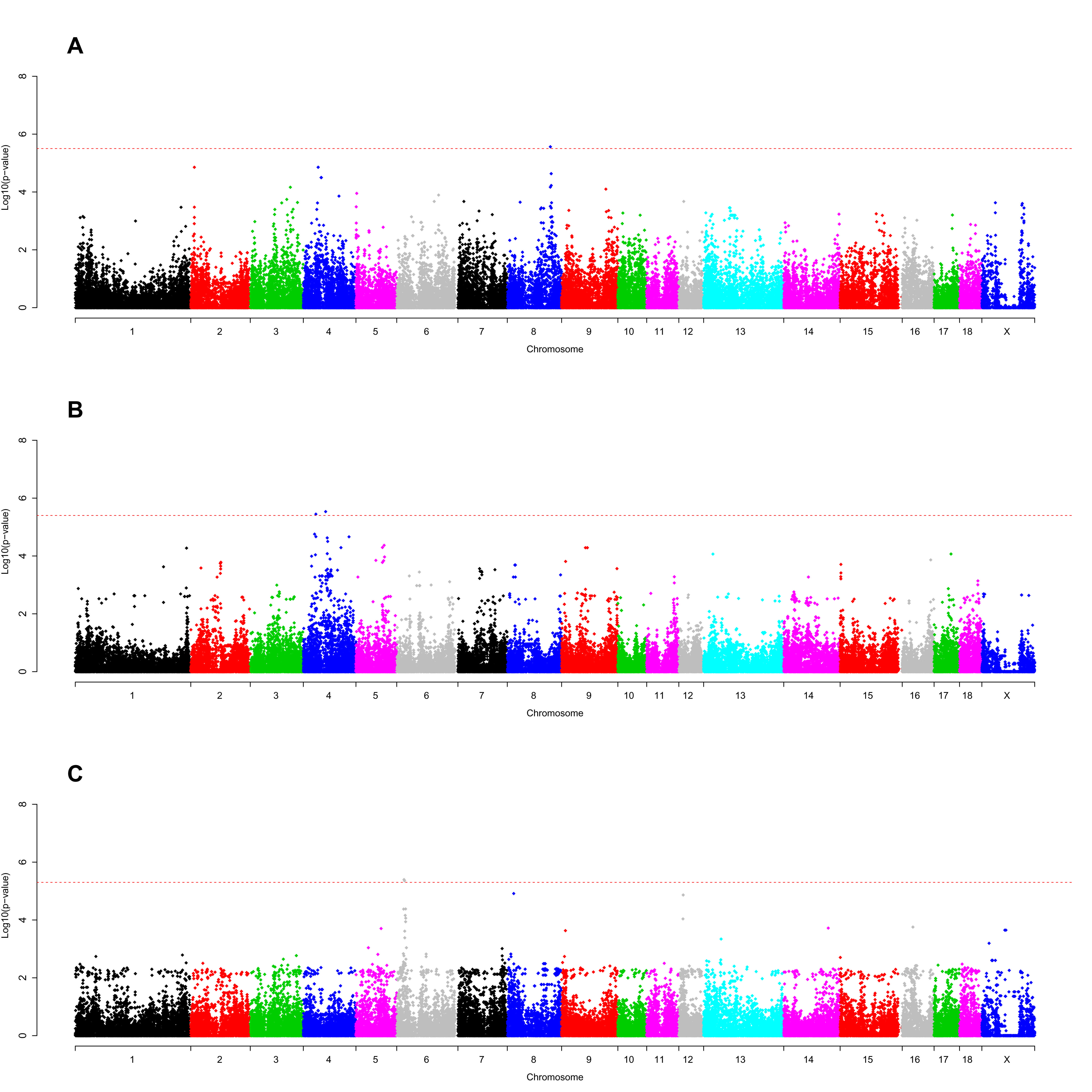

Supplement: Figure S3 — GWAS for ELOVL6 gene expression in backfat, liver and muscle. Association analyses of ELOVL6 expression levels in backfat (A), liver (B) and muscle (C) with genotypes of markers included in the Porcine SNP60 Bead-Chip (Illumina). Positions in Mb are relative to the Sscrofa10.2 assembly of the pig genome. The horizontal, dashed line indicates the genome-wide significance level (FDR-based q-value≤0.1). (TIF) [file pone.0053687.s005.tif]
